# Expression of Major Lipid Raft Protein Raftlin in Chronic Rhinosinusitis with Nasal Polyps in Smoking and Non-Smoking Patients Correlated with Interleukin-17 and Tumor Necrosis Factor-α Levels

**DOI:** 10.3390/biom12091316

**Published:** 2022-09-17

**Authors:** Yu-Tsai Lin, Ming-Hsien Tsai, Yan-Ye Su, Wei-Chih Chen, Shun-Chen Huang, Chih-Yen Chien

**Affiliations:** 1 Department of Otolaryngology, Kaohsiung Chang Gung Memorial Hospital and Chang Gung University College of Medicine, Kaohsiung 833, Taiwan; 2 Kaohsiung Chang Gung Head and Neck Oncology Group, Cancer Center, Kaohsiung Chang Gung Memorial Hospital, Kaohsiung 833, Taiwan; 3 College of Pharmacy and Health Care, Tajen University, Pingtung County 907, Taiwan; 4 Department of Anatomic Pathology, Kaohsiung Chang Gung Memorial Hospital and Chang Gung University, Kaohsiung 833, Taiwan

**Keywords:** Raftlin, chronic rhinosinusitis, nasal polyps, smoking, interleukin-17, tumor necrosis factor-α

## Abstract

Raftlin, as an inflammatory biomarker, has been previously reported in chronic inflammatory diseases. This study investigates the expression of Raftlin in cigarette smokers and in chronic rhinosinusitis with nasal polyps (CRSwNP), as well as evaluating its correlation with interleukin-17 (IL-17) and tumor necrosis factor-α (TNF-α) levels. A total of 30 CRSwNP non-smoking and 16 CRSwNP + SK (smoking) patients undergoing endoscopic sinus surgery were enrolled, while 20 middle turbinate tissue pieces were examined and performed as the control group. In nasal mucosa epithelial staining, Raftlin levels were elevated in the columnar cells and were stained much more intensely in the CRSwNP and CRSwNP + SK groups. Raftlin was located more closely to the apical region of the epithelium in the CRSwNP + SK group; however, the Raftlin levels from whole nasal tissue pieces, according to ELISA data, showed that there was no significant difference between the three different study groups. A positive relationship by Pearson correlation was found between IL-17 or TNF-α levels and Raftlin levels. Taken together, these data indicate that increasing Raftlin expression in columnar cells might involve nasal epithelial remodeling in smokers with CRSwNP.

## 1. Introduction

Chronic rhinosinusitis with nasal polyps (CRSwNP) is an important medical disease, defined by nasal polyps growing in the inflamed tissue of the nasal mucosa as well as the swelling and inflammation of the nasal airway and sinuses, which lasts more than 12 weeks. [1]. Tissue remodeling in chronic rhinosinusitis might occur with provisional or continuous change in the histological composition of the tissues [2,3]. Numerous studies have indicated that sinonasal epithelial tissue remodeling occurs in CRSwNP, depending on the type of inflammation [3,4]. Recently, many reports have pointed out that the biomarkers of chronic inflammation are related to the processing treatments of the paranasal sinuses and nasal polyps [4,5,6], such as Mucin 5AC, Eotaxin, Activin A, Periostin, etc. [6,7,8,9,10].

As a major protein in lipid rafts, Raftlin was identified by Saeki K et al. in 2003 and was realized to play an important role in the transmission and maintenance of lipid rafts. In B-cells, Raftlin was inferred to act as a positive regulator of B-cell antigen receptor (BCR) signal transduction. [11]. In a recent study, Raftlin was revealed as an inflammatory biomarker for several inflammatory diseases. Lee et al., in 2014, demonstrated that Raftlin is related to the severity of sepsis and the dysfunction of endothelial cells, using not only prospective studies but also in vitro/in vivo studies as well, suggesting that Raftlin could be used as a biomarker for determining the severity of sepsis [12]. In a study on Raftlin expression in obstructive sleep apnea (OSA), Bilal et al. revealed Raftlin levels decreased significantly in an OSA group on the third postoperative month [13]. Moreover, in patients with chronic vascular inflammatory disease, such as atherosclerosis, Raftlin levels were shown as highest before treatment and decreased with treatment [14].

Although Raftlin was originally found in B cells, it also played an important role in the regulation of T cell-mediated immune responses [11,15,16]. In addition, Raftlin may be associated with oxidative stress biomarkers malondialdehyde (MDA), total oxidant status (TOS), or catalase (CAT) [14,17], as well as inflammatory biomarkers such as tumor necrosis factor-α (TNF-α) and interleukin-17 (IL-17) [13,16]. Cigarette smoke plays a critical role in the pathogenesis of airway inflammatory diseases. Another report showed that IL-17A levels are markedly and increasingly expressed in CRS patients who smoke cigarettes [18]. However, the expression of Raftlin in CRSwNP concerning smokers remains unclear. In the present study, we investigated Raftlin expression in nasal polyp tissues (with controls and CRSwNP smoker samples) in the Taiwanese population and assessed its relationship with cytokines IL-17 and TNF-α.

## 2. Materials and Methods 

### 2.1. Study Subjects

A total of 66 patients were enrolled from the Department of Otolaryngology in the Kaohsiung Chang Gung Memorial Hospital in Taiwan between September 2017 and July 2020. Nasal polyps from 30 CRSwNP and 16 CRSwNP plus Smoking (CRSwNP + SK) patients, who underwent endoscopic sinus surgery, were assessed. The control group comprised biopsy tissues from the middle turbinate mucosae of 20 subjects who received septo-meatoplasty for relief of nasal obstruction with non-allergic chronic rhinitis, excluding previous sinonasal surgery, nasal tumor, and other sinonasal diseases. The diagnosis of CRSwNP followed the criteria set by the 2020 European Position Paper on Rhinosinusitis and Nasal Polyps (EPOS 2020) [1]. The subjects excluded included: (1) patients who had allergic fungal rhinosinusitis (AFRS) or aspirin-exacerbated respiratory disease (AERD); (2) patients who had immune responses such as ciliary dyskinesia, cystic fibrosis, immunodeficiency, multiple myeloma, or rheumatoid arthritis; (3) patients who had taken immunomodulatory therapies or systemic corticosteroids within 12 weeks before surgery; (4) patients who had asthma; and (5) patients with recurrent CRS. Each CRSwNP patient was evaluated for nasal polyp symptom severity by computed tomography Lund–Mackay scale (LMK-CT) scores [19], with each paranasal sinus scored on a scale of 0 to 2, depending on the level of opacification. The range of total score was 0–24 points. This study was approved by the Institutional Review Board of Chang Gung Medical Foundation (approval numbers 201701016B0 and 201900340B0 on approval date 14 July 2017 and 26 March 2019, respectively).

### 2.2. Measurement of IL-17 and TNF-α

Cytokine IL-17 (also known as IL-17A) and TNF-α measurements were performed simultaneously using the Bio-Plex Human Cytokine Group Assay kit (Bio-Rad Laboratories, Hercules, CA, USA), according to the manufacturer’s instructions. The principle of assay was followed: antibodies specific for human IL-17 or TNF-α coated on a 96-well plate; then, standards and samples were pipetted into the wells and IL-17 or TNF-α was bound to the wells by the immobilized antibody. The wells were washed and biotinylated, anti-human IL-17 or TNF-α antibodies were added. After flushing in triplicate for unbound biotinylated antibody, HRP-conjugated streptavidin was pipetted into the wells. The TMB substrate solution was added to the wells and color developed in proportion to the amount of IL-17 or TNF-α bound. The stop solution changes the color from blue to yellow, and the intensity of the color was measured at 450 nm by Bio-Plex suspension array system, with data analyzed using Bio-Plex Manager software version 6.0 (Bio-Rad Laboratories, Hercules, CA, USA).

### 2.3. Measurement of Human Raftlin

The level of human Raftlin (RFTN1) from nasal mucosae or nasal polyps was assayed using ELISA kits (MBS1600006, MyBioSource, San Diego, CA, USA). This principle of test was based on sandwich enzyme-linked immunosorbent assay (ELISA)-technology following photometric methods with a commercially purchased ELISA kit; an OD absorbance of 450 nm was measured using a microplate reader (BioTek Instruments, Inc., Winooski, VT, USA), and the concentrations of Raftlin were calculated by the following formula: (relative OD 450 nm) = (OD 450 nm of the test well) − (OD 450 nm of the control well).

### 2.4. Tissue Microarray

Tissue microarrays (TMA) were performed using the SIDSCO-TMA70 system (Scientific Integration Design Service Corp., Kaohsiung, Taiwan). The tissue sections were performed using hematoxylin and eosin staining (H&E staining), primary antibody: anti-Raftlin (ab233438, Abcam, Cambridge, UK), anti-IL-17A (ab79056, Abcam) and anti-TNF-α (ab6671, Abcam). Then, tissue sections were incubated with goat anti-rabbit HRP. Rabbit IgG was used as an isotype control (ab37415, Abcam). All slides were mounted with xylene-based mounting medium and scanned at 400× using the Olympus OlyVIA software (Olympus, Tokyo, Japan). The quantitative intensity of staining was analyzed by Image-Pro Plus 6.0 (Media Cybernetics, Rockville, MD, USA) and IHC intensity was represented by mean density, equal to integrated optical density/area of interest, as follows: IHC intensity 0 to 0.15 as weak staining; IHC intensity 0.16 to 0.25 as mild to moderate staining; IHC intensity 0.26 to 0.35 as strong staining; and IHC intensity above 0.35 as very strong staining. The IHC intensity of each slide was determined by at least 3 images.

### 2.5. Statistical Analysis

The statistical analysis and graphing were performed using GraphPad Prism 5.0 software (GraphPad Software Inc., La Jolla, CA, USA). Statistical differences among the means of the study groups were tested by One-Way ANOVA (one-way analysis of variance) with Bartlett’s test for equal variance, while Chi-square testing was used to test for gender data. Pearson’s correlation test was used to measure a linear relationship between two variables. Data were presented as the means and standard error (mean ± SE) and *p* value of 0.05 or less (*p* ≤ 0.05) was considered as statistically significant.

## 3. Results

### 3.1. Patient Demographics

A total of 30 CRSwNP, 16 CRSwNP plus smoking, and 20 control subjects were included in this study. The demographic information for each group, including the presence of atopy, peripheral eosinophil (%), and LMK-CT scores, are described in Table 1. There was no difference in gender or age between all groups, but there were differences in atopy and the LMK-CT scores. The serum IgE level was higher in CRSwNP and CRSwNP + SK patients than in the control group (*p* < 0.001); moreover, there was a higher serum IgE level among the CRSwNP + SK group compared with the CRSwNP group.

### 3.2. Expression of Raftlin in CRSwNP and Smoking

**Figure 1:**
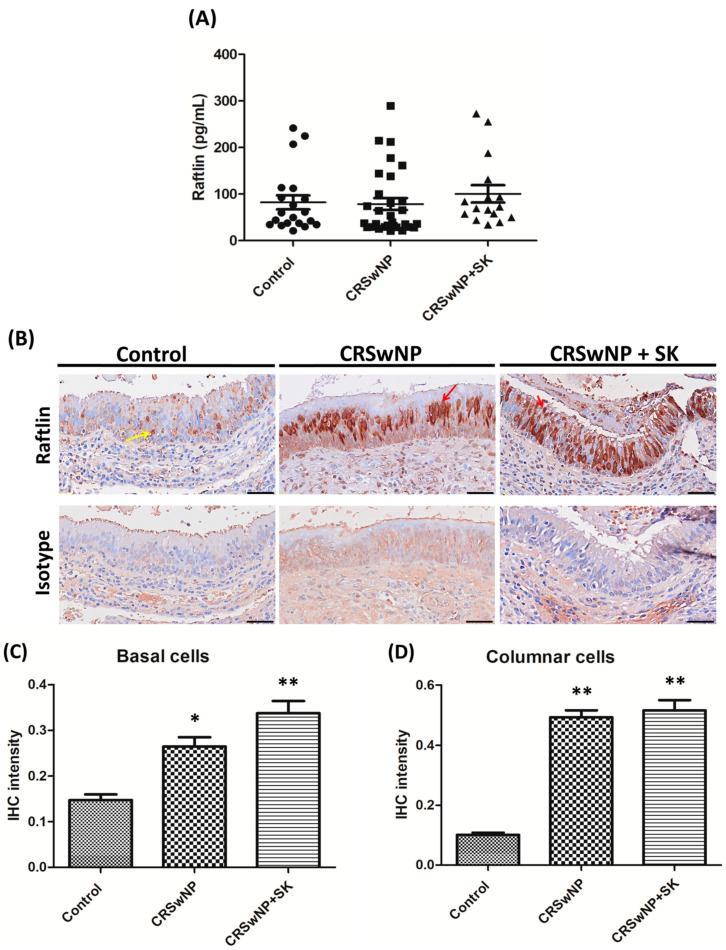
**Theexpressionof Raftlinin nasal tissues.** (**A**) The Raftlin levels from whole nasal tissue pieces in the control (*n* = 20), CRSwNP (*n* = 30), and CRSwNP + SK (*n* = 16) groups were analyzed byELISA assay. (**B**) Representative photomicrograph of nasal epithelium staining of Raftlin expression and its corresponding normal rabbit immunoglobulin G (IgG) as the isotype control for three different study groups. Yellow arrows: basal cells staining and red arrows: columnar cells staining. Scale bar = 50 μm. The quantitative intensity of staining for (**C**) the basal cells and (**D**) columnar cells was measured by Image-Pro Plus 6.0. * *p* < 0.05 vs. control; ** *p* < 0.01 vs. control. SK: smoking.

The expression of Raftlin in whole nasal tissue pieces for all groups was determined using ELISA. The levels of Raftlin in the control, CRSwNP, and CRSwNP + SK groups were 82.2 ± 15.1, 78.6 ± 12.9, and 100.5 ± 18.6 pg/mL, respectively, with no significant differences between the CRSwNP and CRSwNP + SK groups when compared to the control group (Figure 1A). In Figure 1B, which shows nasal mucosa epithelial staining by immunohistochemical staining (IHC), Raftlin expression in the basal cells was stained weakly in the control group. Interestingly, Raftlin expression was elevated in the columnar cells and stained much more intensely among the CRSwNP and CRSwNP + SK groups; besides, Raftlin was located more closely to the apical region of the epithelium in the CRSwNP + SK group. Additionally, the IHC staining intensity of Raftlin is shown in Figure 1C,D. The data indicated that Raftlin intensity in basal cells staining in the CRSwNP and CRSwNP + SK groups was significantly higher than in the control group (Figure 1C; *p* = 0.0079); similarly, the Raftlin intensity in the columnar cell staining was markedly higher among the CRSwNP and CRSwNP + SK groups (Figure 1D; *p* < 0.001).

### 3.3. The Levels of IL-17 and TNF-α Correlated with Raftlin

**Figure 2:**
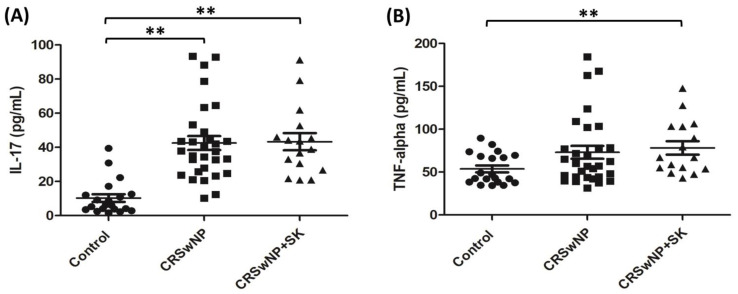

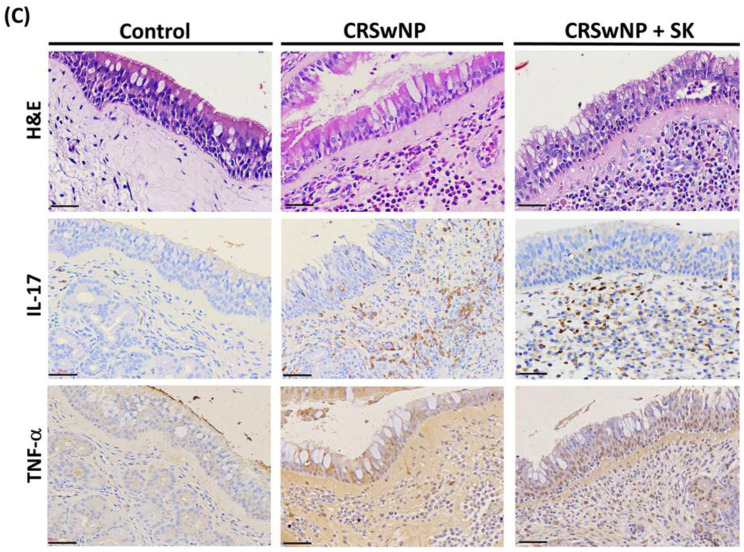
**Theexpressionof IL-17 and TNF-αin nasal tissues.** ELISA data from (**A**) IL-17 and (**B**) TNF-α expression from the whole nasal tissue pieces for the control (*n* = 20), CRSwNP (*n* = 30), and CRSwNP + SK (*n* = 16) groups. ** *p* < 0.01 vs. control. SK: smoking. (**C**) IHC images of IL-17 and TNF-α expression in nasal tissues. The nasal mucosae as a control and nasal polyps as of group CRSwNP and CRSwNP + SK were stained by hematoxylin and eosin (H&E) staining (top panels) and IL-17 (middle panels) and TNF-α (bottom panels) staining. Scale bar = 50 μm.

Raftlin may be markedly related to cytokines, such as IL-17 and TNF-α [13,16]; therefore, the levels of IL-17 and TNF-α for the whole nasal tissue pieces were measured by ELISA, as presented in Figure 2A,B. The levels of IL-17 in the CRSwNP and CRSwNP + SK groups were significantly higher than in the control group (both *p* < 0.001), and there were no significant differences between the CRSwNP and CRSwNP + SK groups (Figure 2A). For TNF-α levels, the amounts of TNF-α in the CRSwNP + SK group were higher than in the control group (*p* = 0.0071), and there were no significant differences among the CRSwNP group as compared to the control group (Figure 2B). In addition, immunohistochemical staining was performed, and the distribution of the IL-17 and TNF-α expression is presented in Figure 2C. In the top panels, the hematoxylin and eosin staining (H&E staining) revealed that there were several changes in the CRSwNP and CRSwNP + SK groups, such as the disruption and shedding of epithelial cells, with increased cytoplasmic vacuolization, large goblet cells, and florid inflammatory cell infiltration in the stroma, yet this was not evident in the control group. The epithelial cells of CRSwNP + SK group were less regularly aligned than those in the CRSwNP group. The second row showed that the inflammatory cells in the CRSwNP and CRSwNP + SK patients significantly expressed IL-17. In the third row, we found that TNF-α was highly expressed, not only in the inflammatory cells, but also in the epithelial basal layer of those patients with CRSwNP and CRSwNP + SK.

**Figure 3:**
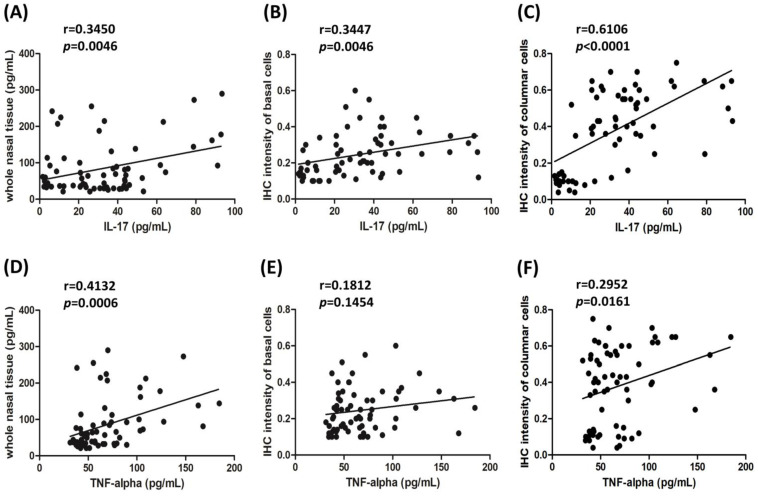
The correlation between IL-17 or TNF-α and the three measurable Raftlin levels (66 samples in total) was analyzed by Pearson R test. (**A**) Correlation between levels of IL-17 and Raftlin from whole nasal tissues, (**B**) between IL-17 and Raftlin from IHC intensity of basal cells, (**C**) between IL-17 and Raftlin from IHC intensity of columnar cells, (**D**) between levels of TNF-α and Raftlin from whole nasal tissues, (**E**) between TNF-α and Raftlin from IHC intensity of basal cells, and (**F**) between TNF-α and Raftlin from IHC intensity of columnar cells in all study subjects. IL: Interleukin; TNF-α: Tumor necrosis factor alpha; *p* < 0.05 was considered a statistically significant difference.

The comparisons between the levels of cytokines IL-17 or TNF-α and Raftlin by Pearson correlation coefficients are shown in Figure 3. The Pearson correlation showed a significant positive relationship between the IL-17 level and all measurable Raftlin levels from the whole nasal tissue pieces was found. The IHC staining intensity of the basal cells and the IHC staining intensity of columnar cells was *r* = 0.3450; 95% CI = 0.1123–0.5418, *r* = 0.3447; 95% CI = 0.1120–0.5416; and r = 0.6106; 95% CI = 0.4324–0.7429, respectively (Figure 3A–C). Moreover, there was also a significantly positive correlation between the TNF-α level and the measurable Raftlin levels from the whole nasal tissue pieces, with the IHC staining intensity of columnar cells being *r* = 0.4132; 95% CI = 0.1902–0.5957; and *r* = 0.2952; 95% CI = 0.05721–0.5015, respectively; however, there was no correlation between the TNF-α level and IHC staining intensity of the basal cells (Figure 3D–F).

## 4. Discussion

A wealth of evidence has indicated that damage to airway epithelium plays a key role in triggering tissue or epithelial remodeling, which is considered an important feature in the many inflammation diseases in the upper and lower airways, such as CRS or asthma [4,5,20,21]. Several reports have investigated the pathophysiological mechanisms of CRS. CRS patients with exposure to cigarettes have higher LMK-CT scores (as disease severity) than non-smokers [22]. Smoking may cause a variance in sinus microbiota and the modification of the physiological and immunological functions of the underlying sinus mucosa. The squamous metaplasia in the olfactory sensory epithelium was observed in CRS patients who were current smokers [22,23,24]. Moreover, some inflammatory biomarkers are associated with the pathogenesis of CRSwNP. A previous reporter indicated that the levels of inflammatory and profibrotic genes, such as profibrotic transforming growth factor beta 1 (TGF-β1) and activin A, as well as downstream TGF-β1 signaling, were present in the stroma of CRSwNP patients. The up-expression of TGF-β1 and activin A from primary nasal epithelial cells (PNECs) in CRSwNP patients was induced by cigarette smoke extract, suggesting that exposure to cigarette smoke might involve airway remodeling [25]. The down-expression of E-prostanoid 2 (EP2) and E-prostanoid 4 (EP4) receptors may be correlated to severe inflammatory reactions in patients with CRSwNP who smoke [26]. Cigarette smoking, causing airway inflammation, is considered to be associated with neutrophil, macrophage, and activated T lymphocyte infiltration and increased cytokine concentrations, such as IL-17A and TNF-α [18,26,27,28,29]. IL-17A and TNF-α are both powerful pro-inflammatory cytokines, which have been reported to be associated with the expression and release of various pro-inflammatory mediators, which lead to tissue damage and epithelial remodeling in the upper and lower airway [28,30,31]. The raft-linking protein Raftlin, as a new inflammatory biomarker, can be responsible for regulating the signal transmission of the B cell antigen receptor (BCR), which plays an important role in the induction of autoimmune and vascular inflammatory responses [11,15]. The present study investigated the major proteins of lipid rafts as Raftlin expression for both non-smoking and smoking Taiwanese CRSwNP patients. Nevertheless, there are some limitations to our clinical study: (1) the difficulty in collecting the health nasal mucosa (IRB limitation), and (2) a low number of patients with CRSwNP (*n* = 3) (this might be due to medical habits in Southern Taiwan).

Our data show that Raftlin levels in whole nasal tissue pieces, analyzed by ELISA assay, showed no significant differences among the CRSwNP and CRSwNP + SK groups when compared to the control group. Interestingly, a recent report from Turkey revealed that nasal polyp tissue Raftlin levels in a CRSwNP group were significantly lower than those of the control group, with no significant differences between CRSwNP and CRSwNP groups regarding asthma [32]. Their data were unlike ours, suggesting that there could exist differences in the profiles of inflammatory biomarkers between CRS patients from different races. Our IHC results found that Raftlin stained much more intensely in the columnar cells among the CRSwNP groups, especially in the CRSwNP + SK group, showing strong staining in the apical region of the epithelium. Furthermore, the Raftlin levels detected in the basal and columnar cells among the CRSwNP and CRSwNP + SK groups were markedly higher than those in the control group. This information indicates that Raftlin expression was elevated in the columnar cells, supporting the notion that Raftlin might be a biomarker for epithelial remodeling in CRSwNP patients.

Raftlin might modulate T-cell receptor-mediated signaling and enhance Th17-mediated autoimmune responses [15] and is considered as an inflammatory biomarker [13,14,17]. Previous reports showed that Raftlin may be related to TNF-α [13,14]. Consequently, we analyzed the IL-17 and TNF-α from whole nasal tissue pieces, and both cytokine levels were markedly higher in the CRSwNP + SK group than in the control group. Besides, our data indicated that the levels of IL-17 and TNF-α significantly correlated with Raftlin levels from whole nasal polyp tissues and in columnar cells. Similar results were reported by some studies, where the level of Raftlin was found to be positively correlated with TNF-α in several chronic inflammatory diseases [13,14]. Collectively, these results suggest that Raftlin translocation may play a role in epithelial remodeling, which is related to IL-17 and TNF-α levels in CRSwNP smoking and non-smoking patients.

Cigarette smoking is a substantial risk factor in the development of upper or lower airway inflammatory diseases, including CRS and asthma [18,33]. A large proportion of patients in the “CRS with smoking” category is found in clinical settings in Taiwan, accounting for around 35~50% of male smokers as being CRS patients [18,34]. Similarly, our data in the present study showed that around 35% of CRSwNP patients were smokers, and 75% of these were males. The other pathogenetic factors for CRSwNP, such as a humid environment, air pollution, as well as ethnic genotype, are distinct in Taiwan, which might help explain the different inflammatory patterns of CRS between Asian and Western populations [35,36]. Only one study has examined the Raftlin in CRSwNP patients in Turkish populations [32], but no previous reports have investigated Raftlin expression in smoking and non-smoking CRSwNP for Taiwanese people. This study is necessary for understanding the effect of Raftlin-associated signaling in the pathogenesis of CRS. In conclusion, our results suggested that increasing Raftlin in columnar cells might involve nasal epithelial remodeling in smoking CRSwNP patients, which correlates with IL-17 and TNF-α levels. Further studies are needed to investigate the therapeutic potential of Raftlin-associated signaling and the mechanism for the reaction between Raftlin and IL-17 or TNF–α in these two subject groups through in vitro or in vivo studies.

## Figures and Tables

**Table 1:** Clinical demographics of subjects in this study.

Demographics	Subjects			
Variables	Control (*n* = 20)	CRSwNP (*n* = 30)	CRSwNP + SK (*n* = 16)	*p* Value
Gender (male), *n* (%)	12 (60.0)	19 (63.3)	12 (75.0)	0.6185
Age (years), Mean ± SE	38.9 ± 2.8	48.3 ± 2.7	49.7 ± 3.5	0.6100
Peripheral eosinophil (%), Mean ± SE	3.2 ± 0.5	3.8 ± 0.5	3.3 ± 0.5	0.5315
Serum IgE level (KU/L), Mean ± SE	78.8 ± 12.8	116.9 ± 20.0	250.9 ± 36.4	<0.001 *
LMK-CT score	-	15.0 ± 0.9	15.6 ± 1.2	0.2686 ^&^
Methodologies used				-
Tissue ELISA (*n*)	20	30	16	
Tissue IHC (*n*)	20	30	16	

* Significance was considered at *p* < 0.05; ^&^ Comparison between CRSwNP and CRSwNP + SK. CRS: chronic rhinosinusitis; NP: Nasal polyps; SK: smoking; IgE: immunoglobulin E; LMK-CT: computed tomography Lund–Mackay; ELISA: enzyme-linked immunosorbent assay; IHC: immunohistochemistry.

## Data Availability

The data presented in this study are available on request from the corresponding author or first author.
